# Environmental Contamination and Hygienic Measures After Feline Calicivirus Field Strain Infections of Cats in a Research Facility

**DOI:** 10.3390/v11100958

**Published:** 2019-10-17

**Authors:** Andrea Monika Spiri, Marina Luisa Meli, Barbara Riond, Imogen Herbert, Margaret J. Hosie, Regina Hofmann-Lehmann

**Affiliations:** 1 Clinical Laboratory, Department of Clinical Diagnostics and Services, and Center for Clinical Studies, Vetsuisse Faculty, University of Zurich, 8057 Zurich, Switzerland; aspiri@vetclinics.uzh.ch (A.M.S.); mmeli@vetclinics.uzh.ch (M.L.M.); briond@vetclinics.uzh.ch (B.R.); 2 Medical Research Council, University of Glasgow Centre for Virus Research, Glasgow G61 1QH, UK; i.herbert.1@research.gla.ac.uk (I.H.); Margaret.Hosie@glasgow.ac.uk (M.J.H.)

**Keywords:** feline calicivirus, cats, virus isolation, environmental testing, RT-qPCR, disinfection, hygienic measures, sodium bicarbonate, shedding, virus survival, veterinary sciences

## Abstract

Feline calicivirus (FCV) can cause painful oral ulcerations, salivation, gingivitis/stomatitis, fever and depression in infected cats; highly virulent virus variants can lead to fatal epizootic outbreaks. Viral transmission occurs directly or indirectly via fomites. The aim of this study was to investigate the presence and viability of FCV in the environment after sequential oronasal infections of specified pathogen-free cats with two FCV field strains in a research facility. Replicating virus was detected in saliva swabs from all ten cats after the first and in four out of ten cats after the second FCV exposure using virus isolation to identify FCV shedders. In the environment, where cleaning, but no disinfection took place, FCV viral RNA was detectable using RT-qPCR on all tested items and surfaces, including cat hair. However, only very limited evidence was found of replicating virus using virus isolation. Viral RNA remained demonstrable for at least 28 days after shedding had ceased in all cats. Disinfection with 5% sodium bicarbonate (and Incidin^TM^ Plus) and barrier measures were effective in that no viral RNA was detectable outside the cat rooms. Our findings are important for any multicat environment to optimize hygienic measures against FCV infection.

## 1. Introduction

Feline calicivirus (FCV) is a common viral pathogen in cats worldwide. The prevalence ranges from 10% up to 70% depending on the cat population sampled [1,2]. Usually, FCV causes painful oral ulcerations, salivation, gingivitis/stomatitis, inappetence, fever and depression with a high morbidity, but low lethality [1,3,4]. However, FCV can occur as a very virulent form, causing severe clinical signs, due to systemic infection and inner organ involvement and high mortality rates up to 60% are reported [5,6,7]. FCV is a non-enveloped RNA virus with a high tenacity that can remain infectious for approximately one month on dry surfaces at room temperature [4], and for weeks at colder temperatures [8,9,10]. Additionally, the stability of FCV is strain-dependent, and common disinfectants do not inactivate FCV [11,12]. Viral transmission occurs via direct cat-to-cat contact or indirectly via fomites (e.g., food bowls, contaminated coats). Reports from animal hospitals encountering outbreaks of virulent FCV infections emphasize that indirect transmission is a key factor in viral spread [5,13,14]. In human hospitals, environmental testing for common human pathogens is routinely performed to assess the hygienic management [15]. In veterinary facilities or research catteries, nobody has so far investigated environmental contamination with FCV.

It was the aim of this study to investigate the presence and viability of FCV in the environment of a research cat facility following two sequential experimental infections of cats with two different FCV field strains. Oropharyngeal shedding of FCV was tested in parallel. The findings of this study are important to optimize the hygienic management of private practices and veterinary hospitals and to prevent accidental, iatrogenic FCV infections in cats.

## 2. Materials and Methods

### 2.1. Animals

In the experiment, 10 male cats, aged 8 weeks and from an approved specified pathogen-free (SPF) facility were used. All cats were group-housed in a confined university facility in four connected rooms (total of ~37.2 m^2^) under ethologically and hygienically ideal conditions [16]. All experiments were conducted according to Swiss law and were approved by the veterinary office of the canton of Zurich (TVB ZH095/15, issued 11 August 2015). After the arrival of the cats and prior to the start of the experiment, each cat was clinically examined, and blood and plasma samples and conjunctival, oropharyngeal, and rectal swabs were collected to verify the cats’ SPF status. These samples were tested for FCV, feline leukemia virus (FeLV), feline immunodeficiency virus (FIV), hemoplasmas, parvovirus (FPV), herpesvirus 1 (FHV-1) and coronavirus (FCoV), as previously described [17,18,19]. The presence of *Bartonella henselae*, *Chlamydia felis*, *Bordetella bronchiseptica* and *Mycoplasma felis* was tested [3,17,18,20]. Feline gammaherpesvirus (FcaGHV) [21] was tested by PCR with modifications as described in Novacco et al., 2019 [22]. In addition, the serum samples were tested for antibodies against feline calicivirus, feline herpesvirus, feline parvovirus and feline coronavirus by immunofluorescence assays as previously described [23,24], and for feline immunodeficiency virus by western blot [25]. All cats tested negative for all of these infections prior to the start of the experiment.

### 2.2. Challenge Viruses

The isolate for the first challenge, FCV 273, originated from a privately-owned Swiss domestic cat. The cat presented with chronic stomatitis/gingivitis, caudal stomatitis and lingual ulcerations. The isolate for the second challenge, FCV 27, originated from a privately-owned Swiss Norwegian Forest cat that was clinically healthy apart from oral ulceration on the hard palate. The sample had been collected for a previous study [3].

Both FCV isolates were obtained from samples collected by Swiss veterinarians using oropharyngeal cytobrushes that were processed using the method described previously [3]. The veterinarians obtained informed consent from the cat owners [3]. All samples were collected as part of a diagnostic workup; no ethical approval was necessary, in compliance with Swiss regulations [26]. The virus was propagated on Crandell-Rees feline kidney (CRFK) cells and passaged three times. After the third passage, cell culture supernatant was harvested when a cytopathic effect (CPE) was clearly visible, but not fully complete and centrifuged at 800× *g* for 3 min. Aliquots of 1 mL were immediately stored at −80 °C. The cell culture supernatants were tested using RT-qPCR and qPCR to confirm the absence of co-infections (FHV-1, FcaGHV, FCoV, FIV, FeLV, FPV, *Mycoplasma felis*, *Bordetella bronchiseptica* and *Chlamydia felis*) as described above.

The capsid sequences of FCV 273 and FCV 27 (KU 747,067 and MN181387, [27]) were compared to each other, and the vaccine strain FCV F9 (M86379) using Geneious 9.8.1 (Geneious, Auckland, New Zealand).

### 2.3. Vaccination and Experimental Infection of the Cats

**Figure 1:**
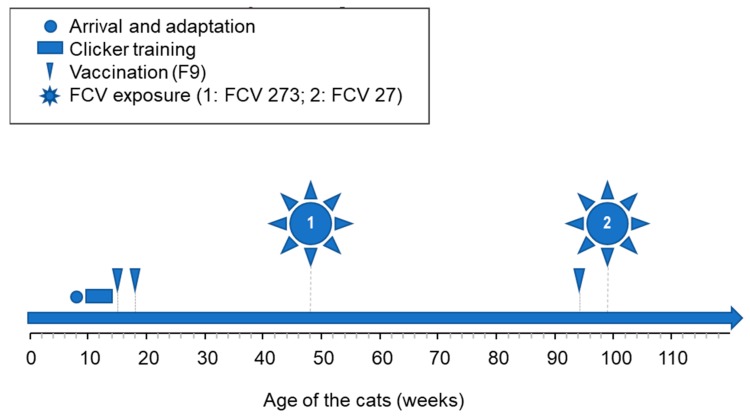
Study design. The cats were first adapted and trained for blood and sample collection. At the age of 15 and 18 weeks, the cats were vaccinated subcutaneously (FCV (feline calicivirus) F9 or placebo vaccine). Seven months later (age of 46 weeks), all cats were challenged using the FCV 273 virus. Eleven months later (94 weeks of age), all cats were revaccinated once (FCV F9 or placebo vaccine) prior to the second FCV challenge with FCV 27 at 99 weeks of age.

The cats were randomly assigned to two groups by a random number generator: Group 1: Cats JJG4, JJG6, JJH3, JJI1, JJI2; and group 2: Cats JJF1, JJG3, JJH2, JJI3, JJI4). The five cats in group 1 were vaccinated subcutaneously at 15 and 18 weeks of age (Figure 1) with a commercially available modified-live virus vaccine (Feligen^®^ CRP, Virbac AG, Glattbrugg, Switzerland). The five cats in group 2 were placebo vaccinated at the same time points with a subcutaneous injection of sterile water. All ten cats were housed together independent of the vaccination status.

All cats were subsequently experimentally infected oronasally with 1 mL of cell culture supernatant containing 1.5 × 10^6^ TCID_50_/mL of the Swiss FCV field strain FCV 273, 7 months after the second vaccination or placebo vaccination (Figure 1).

The cats in group 1 were revaccinated once subcutaneously with Feligen^®^ CRP (Virbac AG), 11 months after the first experimental infection and the cats in group 2 were placebo injected as described previously (Figure 1). Subsequently, one month after revaccination, all cats were oronasally infected with 1 mL of cell culture supernatant (Figure 1) containing 3.2 × 10^6^ TCID_50_/mL of FCV 27.

### 2.4. Sample Collection from Cats and Processing

Oropharyngeal cytobrush samples from the cats were collected on days −4, 3, 9, 15 and weekly thereafter until day 106 after the first experimental FCV infection and on days −4, 3, 9, 15, 22, 42 after the second experimental infection. All samples were processed or adequately stored within 4 h of collection. Cytobrush samples were collected by rolling endocervical sampling brushes (Deltalabs S. L. U., Barcelona, Spain) over the hard palate and the tongue of the cat. The cytobrush from each cat was used for virus isolation on cell culture. For this purpose, the cytobrush was incubated in 700 μL RPMI 1640 (Gibco Life Technologies, Paisley, UK) supplemented with 10% heat-inactivated fetal calf serum (Gibco Life Technologies), 2 mM L-Glutamin (Gibco Life Technologies) and 1x Antibiotic-Antimycotic (Gibco Life Technologies), incubated at 40 °C for 10 min and filtered through a 0.45 μm syringe filter (Filtropur S 0.45 μm, Sarstedt AG and Co. KG, Nümbrecht, Germany). Then, 400 μL of the filtrate were used to inoculate 80–90% confluent CRFK cells in a 24-well plate (TPP Techno Plastic Products AG, Trasadingen, Switzerland). Uninfected cells were run in parallel as negative controls on each plate to check for cross-contamination. FCV causes a characteristic CPE [28], and cultures were observed for CPE daily. As soon as a CPE was observed, or after seven days maximum, supernatants were harvested and frozen at −80 °C until further analysis by FCV specific RT-qPCR for confirmation of presence or absence of FCV viral RNA.

During the first experimental infection, hair samples were collected from one cat (JJH3, group 1) on days 56, 71 and weekly thereafter until day 106; during the second experimental infection, hair samples were collected from all cats once on day 9. The hair was clipped from either the right or left front leg with a trimming machine during the blood collection process in order to visualize the *Vena cephalica*. One half of the hair collected was transferred to a 1.5 mL safe-Lock Eppendorf tube (Eppendorf AG, Hamburg, Germany) and the other half to a micro tube 1.5 mL (Sarstedt AG and Co. KG) containing 300 μL DNA/RNA shield (Zymo Research, Irvine, USA). The hair in the native tube was processed within 4 h for virus isolation as described above. The hair in the DNA/RNA shield was stored at −80 °C until required.

### 2.5. Environmental Sample Collection and Processing

**Figure 2:**
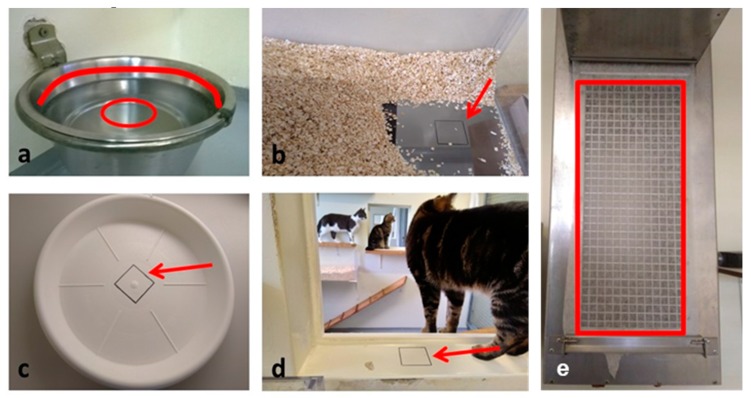
Collection of environmental samples to test for FCV contamination in the cat rooms. (**a**) Water bowl: Four cotton swabs were humidified with sterile phosphate buffered saline (PBS) and rolled over the area above the water level (red line). Four cotton swabs were soaked with the water from the bowl without pre-humidification (red circle). (**b**) Litter tray: Four cotton swabs were humidified with sterile PBS and rolled over the 6 × 6 cm^2^ marked area (red arrow). Two identical litter trays were available for the cats in the facility, and both of them were tested. (**c**) Food bowl: Four cotton swabs were humidified with sterile PBS and rolled over the 6 × 6 cm^2^ marked area (red arrow). Two food bowls were used to serve dry food in the afternoon, the empty bowls were left overnight with the cats, and the sampling was performed the following morning. (**d**) Transfer area between two rooms: Four cotton swabs were humidified with sterile PBS and rolled over the 6 × 6 cm^2^ marked area (red arrow). The ten cats were allowed to move freely between the rooms at all times. (**e**) Ventilation: Six pieces of 1 cm^2^ of the filter paper protecting the ventilation unit from dust and dirt were tested for FCV contamination. The cats had no direct contact with either the ventilation unit or the filter.

The cat facility was tested for FCV contamination after both experimental infections. The items were tested on days −4, 1, 3, 5, 7, 8, 9, 11, 13, 15, 22, 29, 36, 43, 50, 56, 63, 71, 78, 99 and 106 days after the first challenge using direct FCV RT-qPCR and on days 3, 8, 15, 22, 29, 36, 43 and 50 with virus isolation on CRFK cell culture and FCV RT-qPCR from supernatants. The items were also tested on days −4, 3, 8, 9, 15 and 56 after the second challenge using direct FCV RT-qPCR and on days −4, 3, 8, 15, 22, 29, 36, 42, 50 and 56 with virus isolation on CRFK cell culture and FCV RT-qPCR from supernatants. Cotton swabs (M-Budget, Migros Zürich, Switzerland) were collected in quadruplicate from the water bowl (Figure 2a), the water in the water bowl (Figure 2a), the two litter trays (Figure 2b), the two food bowls (Figure 2c) and the area between two connected rooms that were both available to the cats (transfer area) (Figure 2d). The filter that protected the ventilation system in the cat rooms from dust and that was changed daily was also tested for FCV contamination (Figure 2e). The cats did not have direct access to the filter paper of the ventilation, but the filter was within the cat rooms absorbing environmental dust and cat hair. The swabs collected on days 3, 8, 15, 22, 29, 36, 43 and 50 after the first experimental infection and on days −4, 3, 8, 15and 56 after the second experimental infection, were collected as two sets; one set was used for direct RT-qPCR, and the other set was used for virus isolation in cell culture and subsequent RT-qPCR of the supernatants.

**Figure 3:**
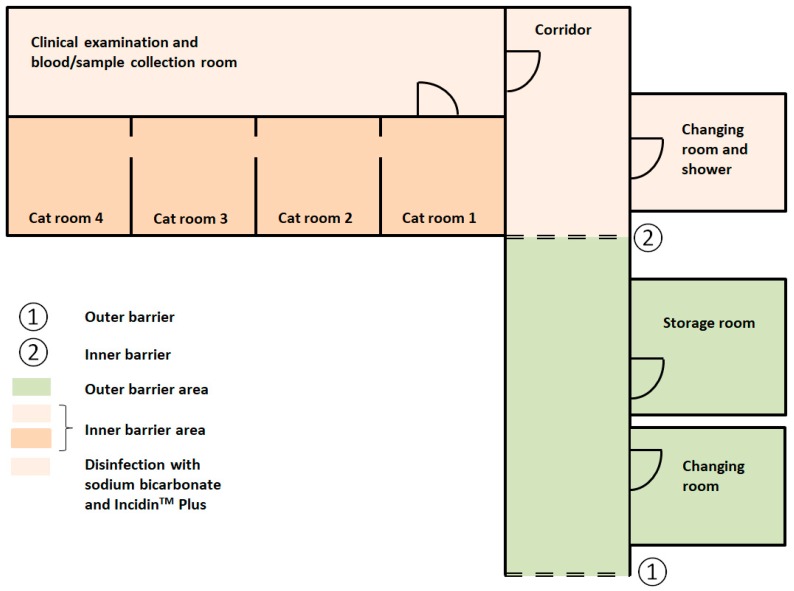
Schematic plot of the cat facility floor plan. The cats were housed in the combined cat rooms 1 to 4 (total 37.2 m^2^; dark orange) within the inner barrier area. The cat rooms were connected by open windows. The cats came into the clinical examination and blood/sample collection room once a day, still within the inner barrier area. They had no access to any other rooms. Only the light orange area was disinfected daily with sodium bicarbonate and Incidin^TM^ Plus. ①: Outer barrier. ②: Inner barrier. Not drawn to scale.

The floor in the food storage room was also sampled (Figure 3) on the same days as the items in the cat rooms. These samples were intended as negative controls, since this room was within the cat facility (within the outer barrier), but outside the inner barrier, separating the FCV-infected cats from the rest of the cat facility area. No cats other than those involved in the FCV experiment were housed in this facility at the same time. All sampled locations apart from the food storage room and the filter of the ventilation were in direct contact with the cats.

The sampling method was adapted from a study investigating human norovirus contamination in a hospital area [15]. For each surface location, an area of 6 × 6 cm^2^ was marked with a waterproof pen, swabs were humified/moistened with sterile 1× phosphate buffered saline (PBS) pH 7.4 (Gibco, Life Technologies) and then rolled over the marked area. The swabs were either placed in a 1.5 mL safe-Lock Eppendorf tube (Eppendorf AG) or in a micro tube 1.5 mL (Sarstedt AG and Co. KG) containing 300 μL DNA/RNA shield (Zymo Research). The shaft of the swab was cut with sterile scissors prior to closing the tube. The protective filter paper of the ventilation system was cut into six pieces of 1 cm^2^ using sterile scissors and each piece was transferred to either a 1.5 mL safe-Lock Eppendorf tube (Eppendorf AG) or to a micro tube 1.5 mL (Sarstedt AG and Co. KG) containing 300 μL DNA/RNA shield (Zymo Research). The set of samples in DNA/RNA shield (Zymo Research) was stored at −80 °C until RNA extraction, and the other set of samples was processed the same day prior to incubation in cell culture. Sampling always took place first thing in the morning before touching any cats, and people handling the samples wore latex gloves. Swabs and protective filter paper pieces dedicated to cell culture were incubated with 200 μL sterile complete RPMI at 40 °C for 10 min. The supernatants from the four swabs or the six pieces from one location were pooled, filtered through a 0.45 μm filter (Filtropur S 0.45 μm, Sarstedt AG and Co. KG) and 400 μL of the filtrate were used to infect 80–90% confluent CRFK cells in a 24 well plate as described above. The cells were checked daily for CPE and as soon as CPE occurred, or after a maximum of seven days, the cell culture supernatant was harvested and frozen at −80 °C until further confirmation by FCV RT-qPCR.

### 2.6. TNA Extraction and PCR

Total nucleic acid (TNA) was extracted from 200 μL of DNA/RNA shield from direct swabs or hair samples or from 200 μL cell culture supernatant using the MagNa Pure LC (Roche Diagnostics AG, Rotkreuz, Switzerland), and the MagNa Pure LC Total Nucleic Acid Isolation Kit (Roche Diagnostics AG) according to the manufacturer’s instructions. For each batch of extractions, negative controls were run in parallel to check for cross-contamination.

Two different real-time RT-qPCR assays (FCV RT-qPCR S1 and S2) were used to detect FCV [3,29,30,31]. The methods had been adapted as follows: Primer and probes were used [3] with the AgPath-ID^TM^ One-step RT-PCR kit (Applied Biosystems, Rotkreuz, Switzerland). The mastermix consisted of 1X RT-PCR buffer, 1.0 μL Array Script reverse transcriptase and AmpliTaq Gold DNA polymerase, 300 nM forward primer [3], 900 nM reverse primer [3], 250 nM probe [3], and nuclease-free water was added to a final volume of 20 μL. All RT-qPCR were run with 5 μL of TNA in a final volume of 25 μL. Positive and negative controls were run in parallel.

### 2.7. Hygienic Measures to Enter and Exit the Cat Facility

To enter the cat facility, the outer barrier and the inner barrier had to be passed (Figure 3). To pass the outer barrier, the following steps were necessary: Undressing of everyday clothes, change of shoes, hand wash with soap and disinfection with Desmanol pure alcoholic hand rub (Schülke, Schülke and Mayr AG, Zürich, Switzerland) and wearing of a single-use overall (Coverall PP, VWR International GmbH, Dietikon, Switzerland). The inner barrier consisted of the following steps: Change of shoes, undressing of the single-use overall, hand disinfection with Sterilium^®^ Virugard (Paul Hartmann AG, Heidenheim, Germany), wearing of a clean cotton fabric overall, a clean pair of socks and a single-use surgical cap. To exit the cat facility, the inner barrier had to be passed firstly and the following steps were necessary: Disposal of the surgical cap to the bio hazard bin (Mauser, Benelux B.V., Oosterhout, The Netherlands), disposal of cotton overall and socks to the wash bin, hand wash with soap, hand disinfection with Sterilium^®^ Virugard (Paul Hartmann AG), disinfection of shoes with Sterilium^®^ Virugard (Paul Hartmann AG), showering of the body with soap, hair wash was only mandatory if the hair was in contact with the cats or anything else within the cat rooms, dressing of single-use overall and change of shoes. To pass the outer barrier, the following steps were necessary: Disposal of single-use overall to the normal bin, changing into everyday clothes and shoes.

### 2.8. Cleaning of the Cat Rooms, the Equipment and the Inner and the Outer Barrier Area

The four connected cat rooms (Figure 3) were dusted daily using a broom, but no disinfection took the place of either the floor or any other material in the cat rooms, including food and water bowls. The bowls were only rinsed with soap after daily use. Obvious stains on the floor (e.g., vomit or urine) were cleaned with water and paper tissues. The cat feces and urine were removed daily from the two litter trays and if necessary new cat litter, Aspen 6 wood shavings (Le Comptoir Des Sciures, Meyzieu, France), was added. Prior to use, the cat litter was autoclaved to prevent the import of pathogens. The cat beds were cleaned with a small brush to remove hair, and, if obviously stained, brought for washing at 60 °C and autoclaving. No additional cleaning or disinfection was performed in-between the two FCV challenges.

In contrast to the cat rooms, the rest of the area within the inner barrier (room for clinical examination and sample collection, corridors, etc.; Figure 3) was cleaned daily with hot water to remove solid dirt and stains and after removing all excess water with a floor squeegee, the floor was disinfected with 5% sodium bicarbonate (kaia.ch/SFT AG, Pratteln, Switzerland) dissolved in hot water as recommended against FCV [32]. Afterwards the excessive water was again removed with a floor squeegee and the disinfection with Incidin^TM^ Plus (Ecolab, Monheim am Rhein, Germany), containing the active agent glucoprotamin, was performed to prevent introduction of any additional pathogens from outside of the facility; glucoprotamin has only a limited virucidal effect, mostly against enveloped viruses [33]. The Incidin^TM^ Plus (Ecolab) was allowed to stay on the floors for at least 15 min before removing it using water and a floor squeegee. All waste that was generated within the inner barrier area was collected in biohazard bins (Mauser), sealed and removed for autoclaving to prevent FCV contamination outside the cat facility.

The used cotton overalls and the dirty cat beds were transported in a leak-proof container to the washing facility outside the cat facility, washed at 60 °C with regular washing powder, dried with a clothes drier, and autoclaved prior to reuse.

### 2.9. Statistics

All data were compiled in Microsoft Excel 2016 and analyzed using GraphPad Prism 8 (Version 8.1.0; GraphPad Prism Software, La Jolla, CA, USA). The Fisher’s exact test was used to test for differences in proportions. The level of significance was a *p* value < 0.05.

## 3. Results

### 3.1. Characterization of the Challenge Viruses FCV 273 and FCV 27

The nucleotide sequences of the capsid genes of the two field isolates, FCV 27 and FCV 273, were compared with each other and with the FCV vaccine strain FCV F9. The sequence identity for FCV 27 and FCV 273 was 77.4%. Compared to FCV F9, FCV 27 and FCV 273 had a sequence identity of 75.8% and 74.7% respectively.

### 3.2. All Cats Shed FCV after the 1^st^ Experimental Infection with FCV 273

Oropharyngeal shedding of FCV was measured using cytobrush material from all ten challenged cats that was tested on CRFK cultures and then performing RT-qPCR on cell culture supernatants. Prior to the FCV challenge, none of the cats was shedding FCV. On days 3 and 9 after the first FCV infection, all samples displayed CPE in cell culture, and the supernatants tested positive for FCV by RT-qPCR (Table 1). Subsequently, CPE was found only in cells incubated with the samples of some cats, but all or most cell culture supernatants still tested positive by FCV RT-qPCR (Table 1). From day 29 onwards, oropharyngeal cytobrushes from six or fewer cats tested FCV positive by RT-qPCR and from day 36 until day 71 only one cat (JJH3) was shedding virus that induced CPE in cell culture. None of the oropharyngeal cytobrush samples tested positive after day 71 following FCV 273 exposure.

### 3.3. FCV RNA Is Present on All Items Tested after the 1^st^ Experimental Infection of Cats with FCV

The presence of FCV RNA was tested by RT-qPCR. FCV positive results were obtained from all items that were either in direct or indirect contact with the cats, but not in the food storage room that was behind the inner barrier (Table 2). Remarkably, also the filter protecting the ventilation that was not in direct contact with the cats and was changed daily tested positive at many time points, but only as long as some cats were shedding FCV.

The highest frequency of positive objects was found early after infection. On days 1 to 13, 6 to 8 out of 8 tested items were FCV positive (Table 2). This decreased to 2 to 4 out of 8 items on days 15 to 56 and finally to 1 to 2 out of 8 on days 63 to 106. No samples were collected between day 106 and day 371, since none of the cats had been shedding as of day 71. Day 371 after the first infection corresponds to day −4 of the second infection. At that time point, all tested items were FCV-negative.

The transfer area between two rooms, the ventilation filter and the two food bowls showed the highest frequency of FCV positivity; 13 to 18 time points out 20 tested after the experimental infection were positive for FCV (Table 2). Interestingly, there was no statistical difference in the frequency of FCV positivity between the three solid dry surfaces in direct contact with the cats (transfer area, food bowls; 73%) and the ventilation filter (aerosol contamination; 70%; p_Fisher_ = 0.7784). In contrast, the water was significantly less frequently positive (15%) compared to the solid dry surfaces (p_Fisher_ < 0.0001). Moreover, the litter trays were also less frequently FCV-positive (20%) compared to the solid dry surfaces (p_Fisher_ < 0.0001). Finally, the water bowl was somewhere in-between the solid dry surfaces and the water (53%).

The FCV viral RNA loads were intermediate to low with cycles of threshold (Ct)-values ranging from 30.9 to 39.9. The lowest Ct-value (30.9; corresponding to the highest load) was observed in a sample from one of the food bowls from day 1 after the first challenge. Ct-values below 35.0, marked in bold (Table 2), were generally observed between days 1 and 13, as well as one sample on day 50.

### 3.4. No Replication Competent FCV Detected on Any of the Tested Items after the 1^st^ FCV Infection

All environmental items were additionally tested for the presence of replicating virus using CRFK cell culture. No virus replication could be detected on CRFK cells as CPE was not observed at any time point for the tested samples. The supernatant from four samples tested weakly positive: The water samples collected on days 3 and 8 (Ct-values 39.3 and 38.3, respectively) and the samples collected from the two food bowls on day 3 (Ct-values 37.2 and 36.2, respectively). All other supernatants were FCV negative by RT-qPCR.

### 3.5. FCV RNA Detected on the Fur of a Cat after the 1^st^ FCV Infection

Clipped hair from either the right or left front leg of one cat (JJH3) collected on days 56, 71 and weekly thereafter until day 106 was analyzed by RT-qPCR and CRFK cell culture (Table 2). This cat had been shedding FCV until day 71. Only on day 85, the hair sample tested positive by direct RT-qPCR (Ct-value 38.7); all other samples were negative, and none of the samples tested in cell culture yielded CPE or RT-qPCR positive supernatant (Table 2). No samples from early infection were available.

### 3.6. Some Cats were Shedding FCV after the 2^nd^ FCV Challenge

On day 3, after the second FCV challenge, four of the ten cats were shedding replicating FCV as determined in cell culture. By day 22, all cats had stopped shedding FCV (Table 3).

### 3.7. No FCV was Found in the Environment and the Cat Fur after the 2^nd^ FCV Infection

The same environmental locations in the cat facility were tested for FCV after the second infection as after the first one by direct RT-qPCR and CRFK cell culture. None of the samples collected at days 3, 8, 9, 15 and 56 after infection tested FCV-positive by either RT-qPCR or virus isolation in CRFK cells, using CPE and RT-qPCR from cell culture supernatants as the readout. Moreover, all hair samples collected on day 9 after the second challenge were found to be FCV negative in direct RT-qPCR, as well as in cell culture as judged by the absence of CPE and negative RT-qPCR results from cell culture supernatants.

## 4. Discussion

This is the first comprehensive study to investigate FCV contamination of a research cat facility following experimental infection of the cats with two FCV field strains. FCV viral RNA was detected at least several times on all items tested. This included items that were in direct contact with the cats, i.e., food and water bowls, water, litter boxes and the cat room floor. However, FCV viral RNA was also detected at the ventilation filter, which was not in direct contact with FCV-shedding cat; this finding indicated that there was the aerosol spread of the virus in environmental dust and cat hair. Although FCV viral RNA was detected on many items, we found no (or only limited) evidence of FCV with replication capacity in the environment, as tested by virus isolation in cell culture. During the entire time course of the study, no FCV was detected outside the inner barrier area. These results are important to understand the potential of FCV for environmental contamination and the effect of optimal hygienic measures.

After the first experimental infection with FCV 273, all ten cats became FCV shedders. Consequently, environmental contamination was found on all tested items in the cat rooms that were in direct or indirect contact with the cats. Subsequently, a decrease in environmental contamination was observed, with fewer items testing positive for FCV RNA over time. Moreover, sampling of the environment in different places was standardized, and samples were analyzed using RT-qPCR. Decreased FCV RNA loads were observed over time. This decrease in environmental contamination reflected the shedding pattern of the cats. Interestingly, the ventilation filter tested positive for FCV as long as the cats were shedding FCV. After the second experimental FCV infection with FCV 27, fewer cats shed FCV and the shedding period was shorter compared to the first experimental infection. Accordingly, the contamination of the environment after the second FCV infection was not as extensive as after the first FCV infection. Indeed, no FCV viral RNA was found on any of the tested environmental items. The lower number of cats shedding FCV and the shorter shedding duration after the second FCV infection were probably not the only reasons for the absence of FCV viral RNA in the environment after the second FCV challenge. During the first infection, only one cat was shedding FCV from day 43 until day 71. Nonetheless, environmental contamination remained detectable up to day 106 after the first FCV infection. Therefore, we hypothesize that the persistence of FCV outside the host could also be strain-dependent. It has been shown that some FCV strains were more resistant to disinfection with different biocides than others [11], and Lee and Gillespie 1972 [34] found differences in the resistance of two different FCV strains to changes in pH. Therefore, further studies with different FCV strains from cats with different clinical signs, including virulent-systemic isolates, will be required to confirm these findings.

We stopped environmental sampling at day 106 until day 371 after the first infection. At day 106, after the first infection, FCV viral RNA was still detected on some items in the cat facility. We can, therefore, not estimate the maximal time span during which FCV might be expected to remain in the environment. However, the persistence of FCV RNA in the environment was at least > 28 days: The last shedding from cats was detected at day 71; no shedding was detected at day 78, and the last positive environmental sample was collected at day 106.

The solid dry surfaces (food bowls, floor in the transfer area and ventilation filter) showed a significantly higher frequency of FCV positivity than the water and the litter trays. This confirms data from a recent study by Buckley et al., 2017 [35], in which it was shown that 30% relative humidity provided a better environment for the survival of FCV than 70% relative humidity [35]. The water bowl tested in our study showed an intermediate frequency of FCV positivity (53%). We assume that the relative humidity for FCV on this item was variable, depending on different water levels in the bowl. It was notable that the frequency of FCV positivity of swabs of the two litter trays was low (19%). Even though FCV is mainly shed via the oral route, there are reports of isolation of FCVs from feline urine [36,37,38]. Limited shedding in urine could account for the limited contamination of the litter tray. On the other hand, FCV shed by the cats could have been absorbed by the litter, which was not tested in this study. In a recent study, it was shown that the type of litter used could influence the environmental spread of FCoV via cat feces and litter boxes [39]. Moreover, the sampling method and the presence of substances, i.e., dust, litter, food on the sample might also influence the extraction efficiency of viral nucleic acids and in turn the result of the subsequent RT-qPCR testing. For further studies on FCV shedding via litter boxes, we suggest testing the cat litter directly and also testing urine and feces samples from FCV-infected cats to detect any FCV shedding via the fecal/urine-route.

During the study, the idea arose to also test the cat hair of infected cats for the persistence of FCV. At that time, 56 days after the first FCV infection, only one cat still tested FCV-positive; therefore, we chose this cat to examine FCV contamination of the hair. FCV RNA was detected on one hair sample collected on day 85, but at no time points thereafter. However, cat JJH3 had stopped shedding FCV after day 71, which could at least partially explain why no more hair samples tested FCV-positive, despite cats regularly licking their hair during grooming. Following the second FCV infection, we collected hair samples early during infection at day 9. However, FCV shedding was diminished after the second FCV infection, and only four cats were shedding FCV at that time. Hair sampling was discontinued after day 9 because none of the hair samples tested FCV RT-qPCR positive. Although none of these hair samples tested FCV RT-qPCR positive, the fur of FCV-infected cats might be expected to be contaminated with FCV.

Although FCV viral RNA was found in abundance in the environment, no replication competent virus was detected using virus isolation; only minimal RT-qPCR signals were found in four of the cell culture supernatants. It has been reported that the number of viral genomes detected, and the presence of infectious viruses do not necessarily correlate [40]. In a study of viral genomes in mineral water, the stability of infectious FCV F9, but not the viral RNA measured by RT-qPCR, was affected by the water temperature [40]. The viral capsid could have been damaged by environmental influences resulting in replication incompetent virions that were nonetheless still detectable by molecular methods [40,41]. This could have also been the case in our study. However, it might also be that the viral loads in this study were too low to initiate efficient virus replication in cell culture; this does not exclude the possibility that the loads might have been sufficient to infect a cat directly. Even so, our findings seem to contrast with current literature. Clay et al., 2005 [8] could recover viable FCV for up to three days from dry surfaces, and in the study of Doultree et al., 1999 [10], FCV stayed replication competent in suspension or in a dry state for up to several weeks, depending on the temperature. However, the latter two studies on FCV stability in the environment used a different approach to that used here; first, the contamination of fomites occurred artificially and second, the FCV laboratory strain F9 was used. The laboratory strain FCV F9 has been used for many years for laboratory and vaccine studies, and therefore, has been propagated in cell culture numerous times. Prolonged culturing of FCV can influence the composition of the virus quasispecies [34]. Radford et al., 1998 [34] demonstrated that the evolution of FCV in cell culture leads to a reduction in quasispecies variability. Additionally, virus neutralization tests showed that FCV isolates directly obtained from cats evolved antigenically over the course of an infection, whereas, isolates passaged in cell culture no longer showed changes in their neutralization pattern [35]. Therefore, prolonged culturing of FCV isolates in cell culture could potentially have influenced the viral behavior in the environment and/or it could have increased the ability of the (recovered) virus to grow in culture. The FCV field strains tested in this study were derived from Swiss domestic pet cats presented to veterinarians with oral ulcerations, gingivitis, stomatitis, fever and depression. The two field strains showed low sequence identity between each other and towards FCV F9, indicating that they are all separate FCV strains [1]. The two field FCV strains were passaged and expanded in cell culture only four to five times before inoculation of the SPF cats in the present study. We suggest that artificial contamination is not comparable with natural contamination via shedding by FCV-infected cats in terms of viral loads and distribution of the contaminant. In particular, the distribution of the contaminant is a challenge for the sampling method in a natural setting. In contrast, in an artificial setting, it is known precisely where the pathogen was placed, and samples can be collected from that location. We tested those places we expected to be contaminated, since each cat had to interact with those places or items on a daily basis. However, in the present study, only a small fraction of the entire possibly contaminated area could be sampled, which could explain the lack of recovery of replicating viruses. The ten cats in this study had a total of 37.2 m^2^ floor space available, and the room was structured in a way that the cats could use it on several levels. We assume that this is more surface space per cat than what isusually provided for cats in clinical environments and rescue shelters. Furthermore, we assume that the sampling method might influence the number of viral particles recovered. Three different methods for the recovery of FCV were compared on wool and nylon carpets in a study by Buckley et al., [35]. On these surfaces, especially, macrofoam-tipped swabbing revealed very low recovery efficiency compared to the bottle extraction and the microbial vacuum method [35]. Although we also used swabbing in our study, we sampled only firm surfaces with cotton swabs. We assume that this is not directly comparable with swab sampling of a carpet.

The hygiene concept consisting of an inner and outer barrier and disinfection measures was successful in preventing the spread of FCV outside of the inner barrier area. This finding emphasized that strict separation of the quarantine area from the rest of the facility is important. Additionally, the choice of disinfectant is also crucial for the success of FCV prevention. Here we used 5% sodium bicarbonate in hot water as a disinfectant against FCV; sodium bicarbonate has been reported to be effective against FCV [42], and was recommended as a safe and inexpensive disinfectant against FCV [32] that is non-toxic for cats and humans. However, it is not effective against many other pathogens [43]. Therefore, we additionally used glucoprotamin to prevent the introduction of other pathogens into the facility. Since glucoprotamin is not effective against FCV, we assume that sodium bicarbonate in combination with barrier measures was effective against spreading of FCV to the outer barrier area. Not only items in direct contact, but also infectious aerosols have to be taken into account when cleaning and disinfecting FCV contaminated areas.

## 5. Conclusions

Veterinarians and animal hospital managers should be aware of the vast contamination potential of FCV, although the persistence of FCV in the environment might be strain-dependent. Each cat suspected to be infected with FCV should be treated as possibly highly infectious for other cats, and appropriate hygienic measurements should be applied. In our study, disinfection with 5% sodium bicarbonate, in combination with barrier measures, worked well.

## Figures and Tables

**Table 1:** Oropharyngeal shedding of FCV after the first experimental infection.

Days Post FCV Challenge I																		
Cat ID	−4	3	9	15	22	29	36	43	50	56	63	71	78	85	92	99	106	371
Cat JJG4																		
Cat JJG6																		
Cat JJH3																		
Cat JJI1																		
Cat JJI2																		
Cat JJF1																		
Cat JJG3																		
Cat JJH2																		
Cat JJI3																		
Cat JJI4																		

Oropharyngeal FCV shedding by the cats was tested using CRFK cell cultures, observation of cytopathic effect (CPE) and FCV RT-qPCR of cell culture supernatants. Black: CPE in cell culture and positive RT-qPCR of cell culture supernatant; Grey: Absence of CPE in cell culture, but positive in RT-qPCR of cell culture supernatant; White: Absence of CPE in cell culture and negative in RT-qPCR of cell culture supernatant.

**Table 2:** Testing for environmental and hair contamination by FCV by RT-PCR after the first experimental infection with FCV 273.

Days after Challenge	−4	1	3	5	7	8	9	11	13	15	22	29	36	43	50	56	63	71	78	85	92	99	106	371
Water bowl			**34.4**	35.9	36.0	**34.1**	**34.2**	**33.5**	36.4	36.0					**33.8**	38.5				nt	nt	38.5		
Water		38.9	37.0	38.6		36.8														nt	nt			
Litter tray 1		37.9				39.0	37.2	38.7	37.9											nt	nt			
Litter tray 2			37.2			**32.3**	38.5													nt	nt			
Transfer area		**34.3**	36.1	35.5	**34.5**	35.4	**33.1**	**33.7**	**33.9**	36.8	36.1	35.3	36.4	35.7	37.4		37.7	38.9		nt	nt	39.4	38.2	
Food bowl 1		**33.4**	35.0	**33.6**	36.4	**34.6**	**34.1**	**34.3**	38.7		37.4	37.3	37.0						39.9	nt	nt		37.9	
Food bowl 2		**30.9**	**33.7**	**33.7**	36.8	**34.9**	36.4	**33.6**	35.4		37.0	38.0		38.6		37.9			38.6	nt	nt			
Ventilation filter		**34.8**		**32.6**	39.2	**34.2**	35.9	36.0	36.0	36.2	36.7	37.4	37.9	36.1		37.1	38.1			nt	nt			
Floor food storage room																				nt	nt			
Hair cat JJH3	nt	nt	nt	nt	nt	nt	nt	nt	nt	nt	nt	nt	nt	nt	nt		nt			38.7				nt

FCV contamination of the listed items in the cat facility and of the hair of cat JJH3 was tested by direct RT-qPCR. Grey: FCV positive; White: FCV negative. nt: Not tested. Numbers in grey boxes indicate the cycle of threshold (Ct-) value. Ct-values below 35.0 are in bold. Framed cells represent the day and localization with additional RT-qPCR positive cell culture supernatants. None of the samples from the storage room, outside the inner barrier and inside the outer barrier, tested positive.

**Table 3:** Oropharyngeal shedding of FCV after the second experimental infection.

Days Post FCV Challenge II						
Cat ID	−4	3	9	15	22	42
Cat JJG4						
Cat JJG6						
Cat JJH3						
Cat JJI1						
Cat JJI2						
Cat JJF1						
Cat JJG3						
Cat JJH2						
Cat JJI3						
Cat JJI4						

The oropharyngeal shedding of FCV from the cats was tested by CRFK cell culture and FCV RT-qPCR of cell culture supernatant. Black: CPE in cell culture and positive RT-qPCR of cell culture supernatant; Grey: Absence of CPE in cell culture, but positive in RT-qPCR of cell culture supernatant; White: Absence of CPE in cell culture and negative in RT-qPCR of cell culture supernatant.
